# Low Resolution Limits and Inaccurate Algorithms Decrease Significantly the Value of Late Loss in Current Drug-Eluting Stent Trials

**DOI:** 10.1155/2012/417250

**Published:** 2012-03-20

**Authors:** Johannes B. Dahm, Frank van Buuren

**Affiliations:** ^1^Department of Cardiology-Angiology, Heart and Vascular Center Neu-Bethlehem, Humboldtallee 6, 37073 Göttingen, Germany; ^2^Genae Core Laboratories, Paleisskaat 24, 2018 Antwerp, Belgium; ^3^Department of Cardiology, Heart & Diabetes Center NRW, Georgstrass, 32545 Bad Oeynhausen, Germany

## Abstract

Quantitative coronary and vascular angiography (QCA resp., QVA) remains the current gold standard for evaluation of restenosis. Late loss as one of the most commonly accepted parameters to highlight efficacy of the various devices has shown high correlation to clinical parameters but, surprisingly, has no impact on the evaluation of the remaining amount of restenostic tissue. The current clinical practice leads to unrealistic late loss calculations. Smaller late loss differences are usually not greater than the inherited resolution limits of QCA, which is especially the case in small differences between the various stents in the drug-eluting stent era. Late loss include additional systematic and random errors, due to the fact that measurements were taken at two different time points including the inherited resolution and calibration limits of QCA on two occasions. Due to the limited value of late loss in discriminating the small differences between the one and other DES, *late lumen area loss* and clearly defined calculation algorithms (e.g., *MLD-relocation*) should be used in future DES studies also to fulfill the more stringent regulatory requirements.

## 1. Text

Endovascular therapy is a rapidly evolving field for the treatment of patients with peripheral arterial occlusive disease (PAOD) or coronary artery disease (CAD), and a magnitude of studies on technical improvements and innovative developments have been published during the last 15 to 20 years. Studies assessing endovascular therapy of peripheral or coronary arteries are tending to hover however on uniformly defined clinical and QCA-derived endpoints, which became clinical practice on the basis of positive correlations to clinical parameters over the last years. Although these QCA-derived parameters were predominantly used to reflect on the neointimal process and to predict restenosis, their values were never evaluated or validated independently. Unfortunately, in order to provide a comparison with the already published studies, most of the subsequently conducted studies used exactly the same QCA-derived study endpoints. Moreover, because the guideline-relevant studies are frequently based on exactly these QCA-derived study endpoints, not only the outcomes of the studies but also the guidelines themselves could therefore be biased by the limitations of these surrogate parameters described in this paper.

In this paper we highlight the algorithm and limitations of late loss as one of the most frequently used QCA-derived surrogate study endpoint in the description of restenosis after interventional lesion treatment and propose other already available QCA-derived parameters as standardized and well defined study endpoint criteria for future restenosis studies presumably based on independently evaluated study endpoint standards formulated by a corresponding consensus committee.

## 2. QCA Quantitative Coronary Analysis

Because intra-arterial angiography remains the current gold standard for the depiction of lesions in peripheral arteries, quantitative coronary/vascular angiography (QCA) has become a trusted tool to evaluate the restenotic process and the relative efficacy of percutaneous endovascular interventions [1]. In order to avoid bias by the interventionist or analysts, validated automatic edge detection became the gold standard in QCA [2–4].

Angiographic parameters derived from QCA such as late loss are common endpoints in stent trials [5–10] although Semeraro and coworkers showed that the location of QCA-based MLD failed to correlate with the location of IVUS-based MLD and the correlation of late loss to “predict” IVUS parameters of restenosis was just moderate [11].

## 3. QCA-Based Estimation of MLD, Diameter Stenosis (% DS), and Late Loss

Measurements of absolute minimum diameters are independent of variations in reference diameter and the extent of reduction in minimum diameter between the immediate postangioplasty and follow-up angiograms defines the extent of vessel wall hyperplasia.

One of the most commonly used angiographic endpoints is late luminal loss, which is the difference between post-procedure and follow-up minimal luminal diameter (MLD).

**Figure 1:**
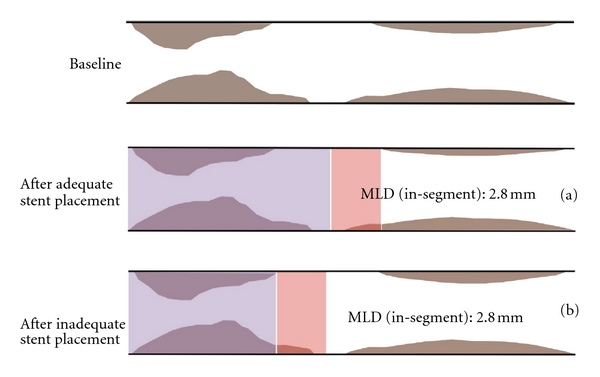
Inappropriate MLD description with similar MLD's (in-segment): (a) in-segment MLD (red area) arising from nontarget lesion tissue in a case after adequate stent placement (purple area) (b) in-segment MLD from in-target-lesion tissue in a case of inappropriate stent landing zone.

It is of utmost importance that late loss does not take the absolute vessel dimensions anatomical or present after the procedure into account (e.g., acute gain), but only the lumen loss during the follow-up period after the procedure. During the evaluation of late loss values, it is therefore mandatory to always look at the MLD values after the final procedure. If a stent has not been implanted adequately (which is probably a fact in almost 35% of the cases) the remaining MLD (e.g., acute gain) remains lower compared to an adequately implanted stent. The late loss calculation however will be lower in cases where the stent was not implanted adequately and where the MLD was lower after the implantation procedure (Figure 1).

In addition, late loss does not take the amount of restenostic tissue into account but exclusively describes the smallest diameter inside the treated segment.

Nevertheless a curvilinear relationship between late loss and the risk of having a target lesion revascularization (TLR) could have been shown in the TAXUS trial [12, 13] and a positive correlation between late loss to binary angiographic restenosis in the SIRIUS study [14].

Thereafter, late loss was used as a gold standard surrogate clinical parameter in predicting restenosis and repeated revascularisation [15, 16].

## 4. Late Loss Differences Are Smaller than the QCA-Resolution Limits

Late loss is a bidimensional parameter defined as the difference between two focal MLD measurements (postprocedural and at followup). As measurements are made at different time points, they are exposed to the inherent 0.2 mm resolution limit of QCA (which barely correspond to the facet of one pixel). In addition, the calibration factor for each MLD measurement is calculated using a different guiding catheter as the reference for each time point. All these factors create systematic and random errors and may explain the poor accuracy and precision of QCA analysis.

In the drug-eluting stent era, absolute late loss values became far smaller than values in the bare metal stent era. Thus, comparing late loss can be relatively reliable when comparing bare metal to drug-eluting stents due to the expected large difference between the devices. Its value can become trivial in trials comparing different types of drug-eluting stents. Indeed, little late loss differences detected after deployment of different drug-eluting stents can still be significant (due to the lower power needed by continuous endpoints to find statistical significance), but their real angiographic and clinical implication can be negligible. When the differences between devices are small (in the order of 0.1–0.4 mm), doubts about the consistency of late loss as a surrogate for the restenotic process will increase. Moreover, if the resolution of QCA is approximately 0.2 mm (e.g., around one pixel), late loss, as derived by two different QCA measurements (after procedure and at followup), can be even more affected by these resolution limits [17].

## 5. MLD and Late Loss Algorithms Are Neither Uniform Nor Clearly Defined

Although late loss is clearly been estimated on the basis of the MLD values (after procedure; at followup), late loss does take into account the location and amount of restenostic tissue within the analysis region and therefore may not be uniform.

**Figure 2:**
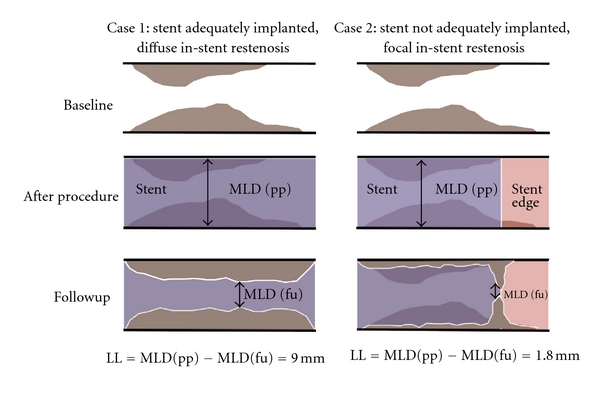
Late loss without association to the true extent of neointimal hyperplasia. Case 2 has a significant higher late loss, due to inadequately implanted stent (purple) at distal stent-edge (red) than case 1, although significant more diffuse neointimal hyperplasia is present in case 1.

**Figure 3:**
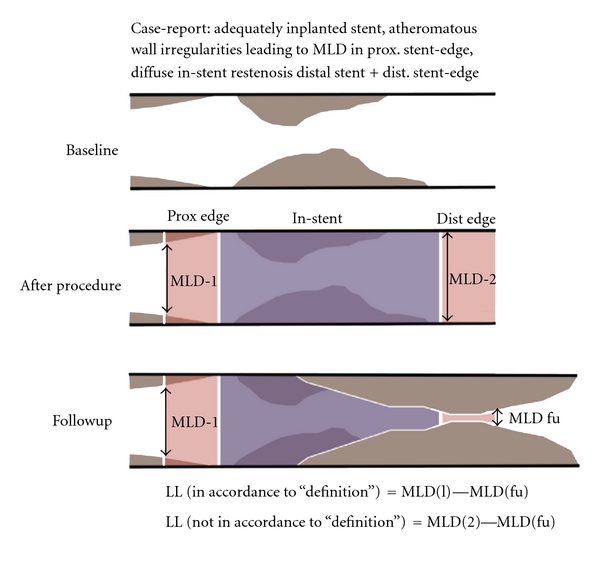
Erroneous late loss algorithm. Although the late loss (= MLD-2−MLD-fu) would reflect the enormous neointimal hyperplasia at the distal stent edge correctly (right red area), the erroneous algorithm/definition of late loss (in-segment) dictates to include the MLD from the opposite stent edge (MLD-1) (left red area) into the equation, leading to an inaccurate lower late loss (not reflecting the true amount of neointimal hyperplasia).

**Figure 4:**
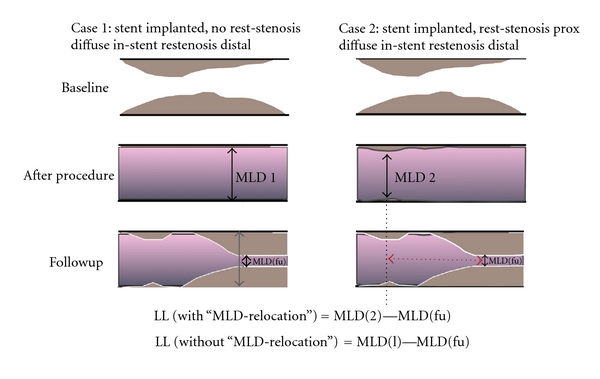
In both cases, a diffuse in-stent restenosis is present. Although the amount of neointimal hyperplasia is exactly the same, the late loss is significantly lower in case 2 due to the remaining MLD-2 after procedure (e.g., restrictive stenosis or nonadequately deployed stent). The red arrow indicates the distance of the *MLD relocation* from MLD-2 to MLD-fu.

1. The measurement of a very low MLD due to a spot restenosis (e.g., in case of a nonadequately implanted stent) as compared to the intermediate-reduced MLD in a uniformly diffuse but not high-degree in-stent restenosis (reflecting a great amount of intimal hyperplasia) does not help to distinguish which device is associated with less intimal hyperplasia (Figure 2).
2. Therefore, the estimation of late loss is independent of the location, length, amount (volume), burden, and distribution of the restenostic tissue and the MLD in case of eccentric vessel appearance may be underestimated by a single monoplane QCA analysis.
3. The measured MLD (in-segment) is not characterized according to its visual and clinical appearance: the remaining target-lesion tissue (in-segment) due to inadequate stent placement is measured as a nontarget lesion tissue from a new atheromatous wall irregularity (in-segment) (Figure 1). Therefore, late loss is calculated regardless of the respective axial location of the MLD along the segments of a stent between postprocedural and follow-up QCA analysis.
4. Late loss is a surrogate parameter for restenosis, although the late loss algorhythm does not necessarily allow its estimation at the site of postprocedural MLD but also elsewhere along the stent [18, 19]. This means, that if stenosis remains somewhere within the treated area or stent (e.g., due to high restrictive areas in the lesion) and restenosis arises in a different area of the stent or stented-segment, late loss is calculated significantly lower as compared with an algorithm calculating late loss exactly at the corresponding place following the procedure. This phenomenon is described as angiographic *MLD relocation*, which occurs frequently and represents an important technical limitation affecting the value of late loss. MLD relocation has the potential to impact adequate calculation of late loss and thus the analysis and interpretation of stent trial results. This commonly occurs in cases where some atherosclerotic tissue remains in the proximal stented segment and in-stent restenosis appears in the distal stent-segment and the stented-segments (proximal and distal) are irrationally calculated as one today: the calculated late loss is outrageously low (Figure 3).
5. Although separate analysis of the QCA derived parameters in the various stent-associated segments became of central importance (i.e., brachytherapy, first drug-eluting stent trials), it later became common clinical practice to summarize the distal and proximal stent-segment as one (*stented-segment*). It became common practice to calculate the late loss of the two opposite segments in one, regardless whether there was MLD remained in the one segment and restenotic tissue appeared in the other segment. This lead to unpredictable errors and severe inaccuracies, which do not reflect the real morphological situation (Figure 4).

## 6. *Late Lumen Area Loss* Effectively Discriminates the Low Differences and Resolution Limits between DES or DEB

**Figure 5:**
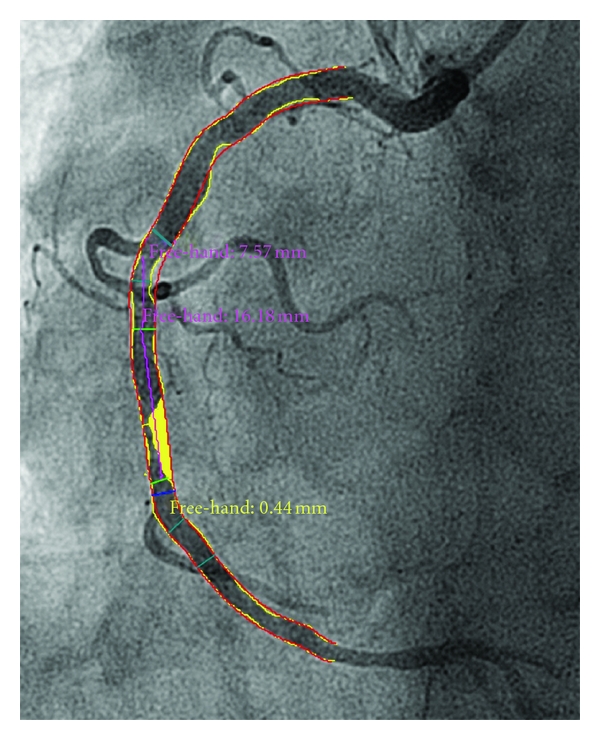
A typical example of a QCA analysis (Medis QAngio, Medis, The Netherlands) of a right coronary artery. *Lumen area* (in mm^2^) is defined as the area around the *lesion area *(yellow area) in a clearly defined vessel segment (in-stent; in-segment) (green lines).

With the use of the following QCA-derived parameters the small differences between two DES or DEB in head-to-head or noninferiority trials can clearly be differentiated. They offer exact information about the true amount and pattern of the restenostic tissue after interventional treatment:

1. *lesion area* (in mm^2^; Figure 5, yellow area;) and *lumen area* defined as area around the *lesion area* in a clearly defined vessel segment (in-stent; in-segment);
2. *late lumen area loss *(in mm^2^) defined as: *late lumen area loss *= *lumen area* (after the final procedure)-*lumen area* (at followup);
3. *MLD relocation* values >3 (5) mm and consecutive assessments of *late lumen diameter loss* calculated on basis of *MLD relocation* should always be documented as such;
4. the *late lumen area loss *and *late lumen diameter loss *should always be assessed in the proximal and distal segment separately.

## 7. Discussion

Although reducing the restenostic process is the central rationale of the majority of coronary devices (i.e., drug-eluting stents or balloons), late loss—as the most frequently used surrogate parameter to highlight efficacy of the various devices surprisingly has no impact on the description of the amount of restenostic tissue and its pattern and distribution.

The consistency of smaller late loss differences is ineffectual and not greater than the inherited resolution limits of QCA which can become clinically evident in DES head-to-head or noninferiority studies since late loss values and differences in these studies became far less significant between the various stents. In addition, late loss includes systematic and random errors, due to the fact that MLD is measured at two different time points, including the inherited resolution and calibration limits of QCA on two occasions.

While using this parameter interventionists must take into account that late loss is calculated regardless of the respective axial location of the MLD along the corresponding segments leading to *MLD-relocation *not only in-stent but also in-segment. This will frequently represent unrealistic late loss calculations. Although these disadvantages may not have an impact on the outcome in the majority of studies (because of being present in the treatment—as well as in the control arm), it is of major interest in smaller studies, or in case of trials with unbalanced or heterogeneous study groups.

In order to eliminate the major disadvantages of the current late loss algorhythm described above, “area” should be used in the late loss algorhythm instead of “diameter” (MLA instead of MLD) leading to the *Late lumen area loss* (LLAL), which has the potential to discriminate the small differences between two DES or DEB in head-to-head or non inferiority trials and offer exact information about the true amount and pattern of the restenostic tissue after interventional treatment.

In addition, greater values of *MLD relocation* and its consequences on *late lumen diameter loss* should always be documented as such and the *late lumen area loss *and *late lumen diameter loss *should always be assessed in the proximal and distal segment separately.

In summary, the invention of late loss area loss (LLAL) as a modification of the well-established and accepted QCA-derived surrogate parameter (LLL) eliminates the major disadvantages of LLL and is an appropriate and reproducible study endpoint for the evaluation of current and future interventional techniques.

## References

[1] Beregi JP, Martin-Teule C, Trogrlic S, Meunier JC, Crochet D (1999). Quantitative angiography: state of the art for peripheral vascular applications. *Journal de Radiologie*.

[2] Serruys PW, Foley DP, de Feyter PJ (1994). *Quantitative Coronary Angiography in Clinical Practice*.

[3] Pentecost MJ, Criqui MH, Dorros G (2003). Guidelines for peripheral percutaneous transluminal angioplasty of the abdominal aorta and lower extremity vessels. *Journal of Vascular and Interventional Radiology*.

[4] Hirsch AT, Haskal ZJ, Hertzer NR (2006). ACC/AHA 2005 guidelines for the management of patients with peripheral arterial disease (lower extremity, renal, mesenteric, and abdominal aortic): executive summary a collaborative report from the American Association for Vascular Surgery/Society for Vascular Surgery, Society for Cardiovascular Angiography and Interventions, Society for Vascular Medicine and Biology, Society of Interventional. *Journal of the American College of Cardiology*.

[5] Mintz GS, Hong MK, Raizner AE (2005). Comparison of quantitative angiographic parameters with the magnitude of neointimal hyperplasia measured by volumetric intravascular ultrasound in patients treated with bare metal and nonpolymeric paclitaxel-coated stents. *American Journal of Cardiology*.

[6] Tsuchida K, García-García HM, Ong ATL (2006). Revisiting late loss and neointimal volumetric measurement in a drug-eluting stent trial: analysis from the SPIRIT FIRST trial. *Catheterization and Cardiovascular Interventions*.

[7] Tsuchida K, Serruys PW, Bruining N (2007). Two-year serial coronary angiographic and intravascular ultrasound analysis of in-stent angiographic late lumen loss and ultrasonic neointimal volume from the TAXUS II trial. *American Journal of Cardiology*.

[8] Kasaoka S, Tobis JM, Akiyama T (1998). Angiographic and intravascular ultrasound predictors of in-stent restenosis. *Journal of the American College of Cardiology*.

[9] De Winter SA, Hamers R, Degertekin M (2004). Retrospective image-based gating of intracoronary ultrasound images for improved quantitative analysis: the intelligate method. *Catheterization and Cardiovascular Interventions*.

[10] Von Birgelen C, De Vrey EA, Mintz GS (1997). ECG-gated three-dimensional intravascular ultrasound: feasibility and reproducibility of the automated analysis of coronary lumen and atherosclerotic plaque dimensions in humans. *Circulation*.

[11] Semeraro O, Agostoni P, Verheye S (2009). Re-examining minimal luminal diameter relocation and quantitative coronary angiography–intravascular ultrasound correlations in stented saphenous vein grafts: methodological insights from the randomised RRISC trial. *EuroIntervention*.

[12] Kuntz RE, Gibson CM, Nobuyoshi M, Baim DS (1993). Generalized model of restenosis after conventional balloon angioplasty, stenting and directional atherectomy. *Journal of the American College of Cardiology*.

[13] Ellis SG, Popma JJ, Lasala JM (2005). Relationship between angiographic late loss and target lesion revascularization after coronary stent implantation: analysis from the TAXUS-IV trial. *Journal of the American College of Cardiology*.

[14] Mauri L, Orav EJ, O’Malley AJ (2005). Relationship of late loss in lumen diameter to coronary restenosis in sirolimus-eluting stents. *Circulation*.

[15] Agostoni P, Valgimigli M, Abbate A, Cosgrave J, Pilati M, Biondi-Zoccai GGL (2006). Is late luminal loss an accurate predictor of the clinical effectiveness of drug-eluting stents in the coronary arteries?. *American Journal of Cardiology*.

[16] Agostoni P, Cosgrave J, Biondi-Zoccai GGL (2007). Angiographic analysis of pattern of late luminal loss in sirolimus- and paclitaxel-eluting stents. *American Journal of Cardiology*.

[17] Agostoni P (2005). Letter regarding article by Mauri et al, "late loss in lumen diameter and binary restenosis for drug-eluting stent comparison". *Circulation*.

[18] Sabaté M, Costa MA, Kozuma K (2000). Methodological and clinical implications of the relocation of the minimal luminal diameter after intracoronary radiation therapy. *Journal of the American College of Cardiology*.

[19] Costa MA, Sabaté M, Angiolillo DJ (2007). Relocation of minimal luminal diameter after bare metal and drug-eluting stent implantation: incidence and impact on angiographic late loss. *Catheterization and Cardiovascular Interventions*.

